# Development of a Theory-Based Nutrition Education Program Targeting Varsity Athletes at a Canadian University

**DOI:** 10.3390/nu18111808

**Published:** 2026-06-04

**Authors:** Jana Daher, Jess Haines, Margo Mountjoy, Dalia El Khoury

**Affiliations:** 1 Department of Family Relations and Applied Nutrition, University of Guelph, Guelph, ON N1G 2W1, Canada; jdaher@uoguelph.ca (J.D.); jahines@uoguelph.ca (J.H.); 2 Department of Family Medicine, McMaster University, Hamilton, ON L8P 1H6, Canada; mmsportdoc@mcmaster.ca

**Keywords:** Theory of Planned Behaviour, dietary supplements, varsity athletes, intention–behaviour gap, nutrition education, behavioral control, intervention development

## Abstract

This paper describes the development and design of *Nutrition for Athletes: A Focus on Dietary Supplements*, an online educational intervention created for varsity athletes at the University of Guelph. Guided by the Theory of Planned Behaviour (TPB), the program aimed to improve athletes’ nutrition and dietary supplement-related knowledge while modifying attitudes, subjective norms, perceived behavioral control, intentions, and behaviors associated with dietary supplement use. Formative research with the target population revealed widespread misconceptions, strong social influences, and high perceived benefits of supplement use, which highlighted the need for an intervention focused on reshaping underlying beliefs. The program covered topics related to sports nutrition, hydration, dietary supplements, and risks of supplement use, and was integrated into the university’s learning management system. Each unit was mapped onto relevant TPB constructs, with an emphasis on correcting inaccurate beliefs and promoting a food-first approach. The program’s effectiveness was evaluated through pre- and post-intervention questionnaires assessing knowledge and TPB constructs. This paper outlines the theoretical framework, development process, and content structure of the intervention, and presents a model that can be replicated in future educational programs.

## 1. Introduction

There is an extensive body of research which suggests that the development of interventions informed by theory is necessary to evoke behavior change and deliver population health outcomes [1,2,3,4,5]. The effectiveness of theory-informed interventions has been debated by some researchers [6,7]. There are a number of reasons underpinning the debate. Theory is often poorly applied in design as well as evaluation of behavior-change interventions. This could be due to selecting a theory that is inappropriate to the context of the intervention, failing to follow the necessary steps in the design process, targeting only a few theoretical constructs, or loosely referring to the theory rather than applying it rigorously [8]. Icek Ajzen, the founder of the Theory of Planned Behaviour (TPB), argues that negative outcomes of poorly conducted studies are often misinterpreted as evidence against the theory [9].

The added value of theory-based interventions includes: (1) ability to measure intervention outcomes and change in theoretical constructs, (2) identification of which components work and which do not, which helps refine and improve interventions, (3) description of causal factors of change and understanding how the behaviour changes, (4) clarification whether interventions fail due to the construct not being influenced or by not having an effect on behaviour, (5) consolidation of a cumulative knowledge of how behaviour changes across different contexts and target populations, (6) offering an opportunity to test and develop theories, ultimately optimizing behaviour-change interventions [8,10,11,12,13,14].

Despite the plethora of evidence supporting the effectiveness of theory-based interventions [15], the use of theory remains limited, where a significant number of studies still lack a theoretical framework [8]. In a narrative review on interventions targeting dietary supplement practices up to 2021, 22 out of 25 articles were atheoretical [16]. However, a scoping review about interventions designed to improve women’s diets in low- and middle-income calories by researchers from Cornell University, stated that articles conducted after 2020 were more likely to be based on a theory of change [17].

A key issue in the literature is that many published intervention studies lack sufficient detail, making it difficult to understand what was implemented, what mechanisms were proposed, and how the intervention could be replicated [12,18]. Unclear and missing intervention descriptions can lead to unintended variations in supposedly identical interventions, making it challenging to identify key details and ultimately complicating the interpretation of inconsistent results [19].

The purpose of this paper is to describe the development, design, and content of the *“Nutrition for Athletes: A Focus on Dietary Supplements”* program, an online education intervention that aimed to improve the University of Guelph’s varsity athletes’ knowledge, intentions, related determinants, and behaviors related to nutrition and dietary supplements. The Theory of Planned Behaviour was used as a methodological framework for program design; therefore, the article will also delve into how the program addressed the Theory of Planned Behaviour constructs.

## 2. Rationale

Studies have found that athletes often have limited knowledge of nutrition, particularly regarding dietary supplements [20,21]. Several researchers have reported a significant deficit in nutritional knowledge among this group and have emphasized the need for educational programs to improve athletes’ understandings and attitudes toward dietary supplement use [22]. In addition, they were found to often rely on unreliable resources for sports nutrition information, including friends and family, teammates, coaches, etc. [23]. A recent study has found that most of the participating athletes heavily relied on their coaches and social media as key information sources regarding dietary supplements. This raises concerns, especially that athletes remain a major part of dietary supplement users [24].

The efficacy and safety of dietary supplements, especially those claimed to enhance performance, are questioned. Most of these claims are often not supported by sound evidence, in addition to the absence of a defined standard for the quality of evidence required to support such claims [25]. However, due to the limited regulatory oversight in the dietary supplement industry, many products still use misleading marketing strategies that advertise unsupported health claims [26].

Furthermore, athletes consuming dietary supplements, especially “performance-enhancing” ones, are at a higher risk of inadvertent doping, due to potential contamination with prohibited substances, putting their careers at risk [27,28,29]. Around 48% of dietary supplement manufacturers and importers had their licenses suspended by the Canadian government due to premises, staff, licenses, sanitation, and quality assurance [26]. Current data have reported that consumers are unaware of the harmful potential of dietary supplements [23,30,31].

Recently, there has been a rise in aggressive marketing of sports supplements, particularly through social media, that has further contributed to misconceptions around their necessity and replaced advice provided by professionals [32,33]. This is particularly concerning for younger athletes, including those at the varsity level, who are often at a formative stage of identity development and are more likely to be influenced by their peers. This desire to align with perceived norms in athletic environments, coupled with a strong drive to try things that promise a competitive advantage, can lead them to adopt supplement use practices without critically evaluating their safety or effectiveness [34].

Given these risks associated with poor nutritional knowledge and dietary supplement practices, educational interventions are essential to provide athletes with accurate information and raise awareness of the potential dangers, especially among young athletes, who are at a critical period of development that establishes lifelong health practices [35]. The “Nutrition for Athletes: A Focus on Dietary Supplements” program targets varsity athletes, a target population that is under-researched, despite their widespread use of dietary supplements [16].

## 3. Theoretical Framework

### 3.1. Theory of Planned Behaviour

**Figure 1:**
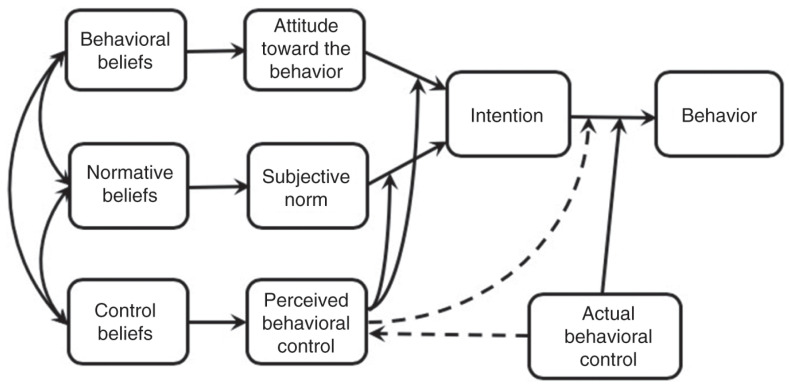
Schematic representation of the theory of planned behaviour.

The Theory of Planned Behaviour was used as a framework to design, develop, and evaluate this intervention program (Figure 1). An extension of the Theory of Reasoned Action, the Theory of Planned Behaviour posits that the proximal determinant of behaviour are intentions [36,37]. These intentions are influenced by three main constructs: attitudes, subjective norms, and perceived behavioral control. When these factors are positive, an individual is more likely to develop strong intentions, and ultimately perform a behaviour [38].

#### 3.1.1. Attitudes and Behavioral Beliefs

Attitudes are how favorably or unfavorably one views a certain behaviour. An attitude is considered a function of a behavioral belief about the consequences or outcomes associated with a behavior [9]. Therefore, behavioral beliefs are assumed to be responsible for the formation of a positive or a negative attitude. For example, a pregnant woman might believe that breastfeeding (behavior) is unlikely to strengthen her baby’s immune system (positive consequence), and that it is likely that breastfeeding (behavior) will lead to breast ptosis (negative consequence). Since this woman thinks that she will experience a negative consequence, she will likely hold a negative attitude towards breastfeeding and is therefore less likely to breastfeed.

#### 3.1.2. Subjective Norm and Normative Beliefs

Based on normative beliefs, subjective norm is the perceived social pressure to engage or not engage in a behaviour. Normative beliefs are beliefs about the expectations and behaviours of our social circle or important others, such as family, friends, spouse, role models, etc. It is what forms our perceived social pressure, referred to as subjective norm. Subjective norms consist of injunctive norms, the perceived pressure from others to perform a behaviour (or not), and the descriptive norms, which are the perceptions of whether significant social referents perform the given behaviour or not [39]. However, it is important to note that the influence of subjective norms varies based on whether the person cares to adhere to the expectations of their society or not [36]. For instance, if a young person perceives that their friends are extremely likely to approve of them engaging in drinking behaviour, but that their family is unlikely to approve, they are more likely to follow the influence of the group they feel more pressure or motivation to please.

#### 3.1.3. Control Beliefs

Perceived behavioral control is one’s belief in their ability to carry out a behaviour. Control beliefs refer to an individual’s perception of factors that may help or hinder their ability to perform a behaviour. These beliefs, combined with the perceived power of each factor, shape a person’s perceived behavioral control [40]. Factors include essential skills needed to perform a behaviour, access to resources and social support, among others. These factors allow an individual to evaluate their ability to perform a behaviour [36]. For example, a varsity athlete might be extremely confident that they have the required cooking skills and resources (facilitating factor) to cook healthy food. However, they might also believe that time constraints (inhibiting factor) impede their ability to cook healthy food. While both factors contribute to their control beliefs, the prevailing perceived behavioral control will depend on the factor that they perceive exerts more influence. Perceived behavioral control serves as a direct predictor of behaviour, to the extent that the measure matches actual control [12].

The Theory of Planned Behaviour has been extensively used as a framework for the design and evaluation of behavioral interventions and is one of the most widely adopted social cognitive models for the prediction of behaviour. The effectiveness of Theory of Planned Behaviour-informed interventions has been consistently demonstrated by numerous reviews [12,41,42,43]. A three-level meta-analysis that investigated the effectiveness of behaviour-change interventions based on the Theory of Planned Behaviour showed significant effects, although the effect sizes varied across different behavioral domains [44]. Furthermore, the theory distinguishes between individuals who have the intentions to perform a certain behaviour but lack behavioral control and those who are not motivated to perform a behaviour (i.e., have weak intentions) [9]. This distinction helps focus interventions on the actual barriers of behaviour change.

Therefore, it is critical to identify the two distinct focuses of a Theory of Planned Behaviour-based intervention: motivating individuals who lack or have weak intentions to perform a given behaviour; or enabling those who are not able to act on their already-existing intentions [9]. If our target population does not intend to perform a behaviour, the Theory of Planned Behaviour identifies the beliefs that have to be changed to modify intentions. However, the theory does not (and was not designed) to recommend intervention techniques required to bring about this change. These approaches typically depend on the specific scope of each intervention [9]. Changes in attitudes, subjective norms, and perceived behavioral control should be reflected by changes in corresponding beliefs, ultimately modifying intentions. For a target population that already has favourable intentions, the intervention that must establish ways to initiate and maintain the desired behavior is needed to close the intention–behaviour gap [45]. Additionally, for intentions to translate into the desired behaviour, the necessary resources must be available, and any barriers that hinder action should be eliminated [9].

Therefore, given its well-established applicability and effectiveness, we selected the Theory of Planned Behaviour to guide the development and evaluation of our online interventional education program. However, prior to developing the program, it was necessary to identify the target behaviour and population, and conduct preliminary research that would help us decide what constructs our intervention should target.

## 4. Intervention Development

### 4.1. Target Behaviour and Population

Our intervention target behaviour was the use of dietary supplements. The context was generalized to include all relevant contexts (e.g., at home, at the gym, at the university, etc.). Our target population was varsity athletes at the University of Guelph, with no recruitment restrictions based on team affiliation, sport type, or academic level.

### 4.2. Formative Research

Since beliefs change from one population to another and over time in the same population, the information relied on to develop the intervention was derived through formative research from varsity athletes at the University of Guelph, the target population for this study. The goal of our formative research was to understand whether our intervention should address existing beliefs or create new ones, through investigating nutrition and dietary supplementation knowledge and practices. This information was collected using a Theory of Planned Behaviour-based questionnaire, alongside a supplement use questionnaire, which assessed Theory of Planned Behaviour constructs related to dietary supplement use, supplementation practices, sources of information, and other relevant factors. The data were gathered from students and varsity athletes at the University of Guelph a year before our intervention development, through two research projects by Dr. Dalia El Khoury, the supervisor of this project, and her research team [26,46].

The data revealed several key findings that helped shape the direction of our intervention program. First, students who used dietary supplements were found to believe that dietary supplements improve their performance, appearance, and health, and that dietary supplements are safe to use. They believed that their social circle used dietary supplements and encouraged them to do so. They also believed that their healthcare professionals thought they should use dietary supplements. Their perceived behavioral control to use dietary supplements was high, an expected finding considering their existing dietary supplement use behaviour. This research investigating dietary supplementation practices among varsity athletes at the University of Guelph revealed that around 60% of the participants reported having consumed dietary supplements in the past 6 months [26]. While non-users generally held opposite beliefs, these findings were alarming considering the high prevalence of dietary supplement use among varsity athletes.

This information guided the development of our program and highlighted the importance of addressing the underlying beliefs of our target population to effectively modify their attitudes, subjective norms, perceived behavioral control, and intentions. It also helped us understand that our target population does not have existing intentions to avoid using dietary supplements; therefore, our focus should not be on barriers hindering those intentions, but on modifying beliefs that encourage them to engage in dietary supplement use, most of which are not based on scientific evidence.

### 4.3. Identifying Objectives and Outcomes

Our intervention outcomes and objectives were based on the findings of the formative assessment, which indicated a need to change existing beliefs that encourage them to engage in dietary supplement use. This also included identifying how the determinants of behaviour should be targeted. While the intervention aimed to target all the Theory of Planned Behaviour constructs, the educational focus of the intervention placed an emphasis on addressing behavioral beliefs (i.e., attitudes). Below is an overview of the intervention’s outcomes and corresponding objectives.

- Outcome 1: Decrease athletes’ attitudes towards using dietary supplements.
  - Objective 1a: Decrease athletes’ beliefs towards the performance-enhancement potential of supplements.
  - Objective 1b: Improve athletes’ nutritional knowledge.
  - Objective 1c: Improve athletes’ knowledge about the risks associated with dietary supplement use.
- Outcome 2: Improve athletes’ subjective norms by reducing perceived social pressure to use dietary supplements.
  - Objective 2a: Increase athletes’ perception that teammates, coaches, and mentor varsity athletes are taking dietary supplements only if needed and under guidance (after diet alone has been considered).
  - Objective 2b: Increase athletes’ perception that teammates, coaches and mentor varsity athletes support them avoiding dietary supplements except if needed and under guidance.
- Outcome 3: Increase athletes’ perceived behavioral control to avoid using dietary supplements.
  - Objective 3a: Improve athletes’ readiness to consult a healthcare professional (i.e., sports doctor; registered dietitian) when facing behavioral, cognitive, emotional and social barriers impacting their decision-making process with respect to dietary supplements.
- Outcome 4: Increase athlete’s intention to take nutrients needed from diet first before considering dietary supplements.
- Outcome 5: Decrease athletes’ dietary supplement use.

### 4.4. Content Development

**Figure 2:**
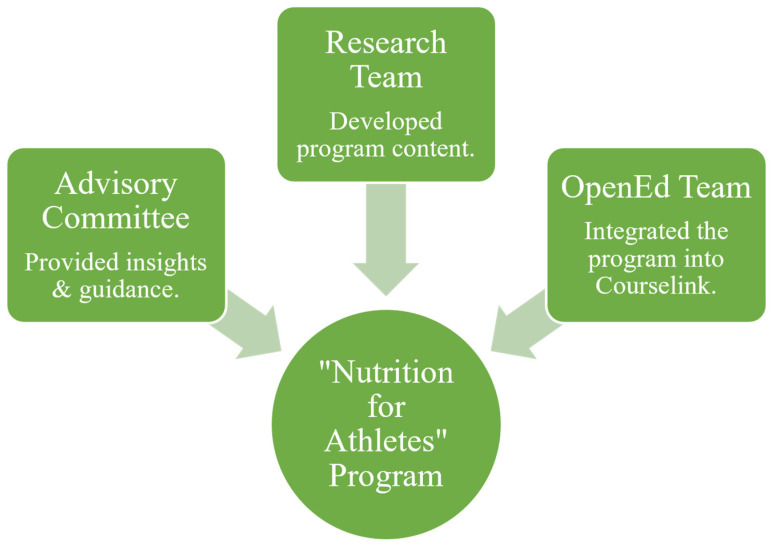
Key contributors to the development of the program.

The content development and online integration of the program was a collaborative effort between Dr. Dalia El Khoury’s research team at the University of Guelph, an advisory committee, and the Open Learning and Educational Support (OpenEd) team. The research team consisted of Dr. El Khoury, an Associate Professor at the University of Guelph, Moriah Mallick, an undergraduate research assistant, and Jana Daher, a PhD candidate in Applied Human Nutrition at the University of Guelph. An academic advisory committee provided insights and guidance throughout the program development. The OpenEd team helped integrate the program into CourseLink, the University of Guelph’s learning management system (Figure 2).

The selection and organization of the program content were informed by multiple factors. These included findings from the formative research conducted within the target population, the current literature on sports nutrition and dietary supplement use among athletes, and the Theory of Planned Behavior constructs targeted by the intervention. The research team selected topics that addressed the most common misconceptions, beliefs, and practices identified among varsity athletes, particularly those related to the perceived benefits, safety, and necessity of dietary supplements. The modules were intentionally organized in a progressive sequence, beginning with foundational concepts in sports nutrition and hydration before introducing dietary supplements and the potential risks associated with their use. The program was designed as a self-paced online intervention that participants could complete over multiple sessions. Each unit’s reading time was around 15–20 min.

#### 4.4.1. Topics and Learning Outcomes

After holding several discussion sessions, the research team decided to divide the program into four separate units, each focusing on a distinct topic.

1. Unit One: Nutrition in Sports

**Figure 3:**
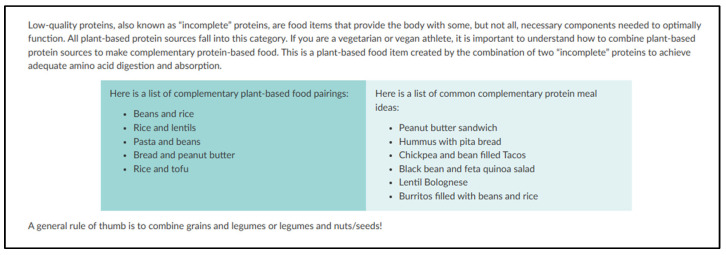
A snapshot from the “Nutrition in Sports” unit.

The first unit introduced the foundational concepts of sports nutrition and explained the role of macronutrients, as well as the intake recommendations for athletes. The unit also provided examples of good sources of each macronutrient in addition to meal ideas. It also focused on the importance of nutrient timing, what they should eat—and avoid—before, during, and after exercise (Figure 3).

The learning outcomes of the “Nutrition in Sports” unit were to:

- Describe the impact and importance of adequate nutrition in sports.
- Explain what factors influence nutritional intake relating to exercise.
- Time your nutritional intake to enhance athletic performance and prevent discomfort.
- Explain the importance of adequately fueling your body.

The Theory of Planned Behaviour constructs addressed in Unit 1 were attitudes and perceived behavioral control.

2. Unit Two: Water and Hydration in Sports

**Figure 4:**
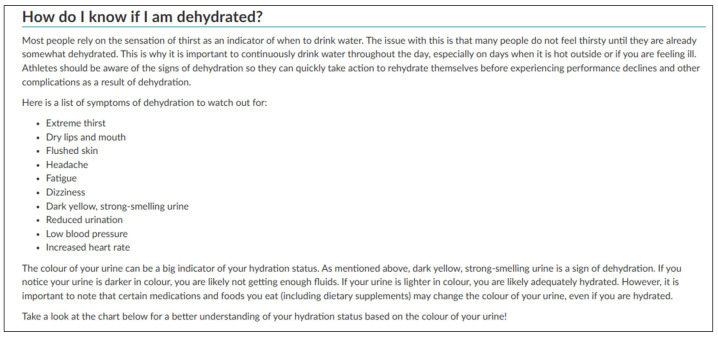
A snapshot from the “Hydration in Sports” unit.

The second unit focused on the importance of adequate hydration in athletic performance and provided practical advice on how to recognize dehydration through physical signs and symptoms. The unit included a urine color chart with a detailed description to monitor their hydration status. Effective steps to prevent dehydration before, during, and after exercise were thoroughly discussed. This unit identified fluids that should be avoided as poor hydration equivalents. Additionally, it provided a simple, easy-to-make electrolyte drink recipe that athletes can prepare after training to replenish lost fluids and electrolytes (Figure 4).

- The learning outcomes of the “Water and Hydration in Sports” unit were to:
- Describe the importance of adequate hydration in sports.
- Identify the signs of dehydration.
- Implement adequate hydration practices before, during, and post-exercise.
- Explain factors that influence fluid needs.

The Theory of Planned Behaviour constructs addressed in Unit 2 were attitudes and perceived behavioral control.

3. Unit Three: Dietary Supplements

This unit introduced the different types of dietary supplements and focused on sports supplements often consumed by athletes, such as protein and amino acid supplements, creatine, caffeine, sports drinks, and sports bars. The unit highlighted the importance of consulting a healthcare professional before using dietary supplements. It covered the circumstances under which athletes may benefit from supplements and emphasized that the claims made by supplement manufacturers are not always based on scientific evidence. Lastly, we explored the effectiveness and potential side effects of dietary supplements commonly consumed by athletes.

The learning outcomes of the “Dietary Supplements” unit were to:

- Identify the purpose of dietary supplements.
- Describe the effects certain dietary supplements have on athletic performance.
- Recognize that dietary supplement consumption is not always necessary.

The Theory of Planned Behaviour constructs addressed in Unit 3 were attitudes, injunctive norms, descriptive norms, and perceived behavioral control.

4. Unit Four: Risks Associated with Dietary Supplement Use

**Figure 5:**
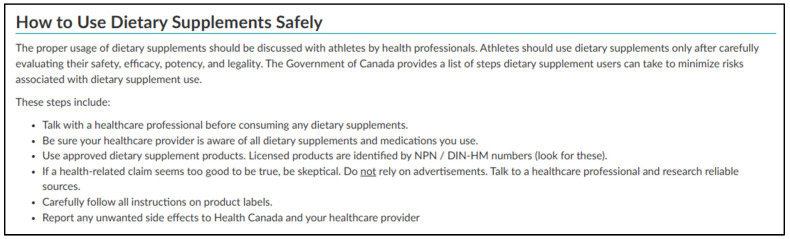
A snapshot from the “Risks Associated with Dietary Supplement Use” unit.

This unit consisted of four subsections: (1) doping and fair play in sports; (2) safety, quality, and marketing of dietary supplements; (3) risks associated with dietary supplement use; (4) how to use dietary supplements safely. The risks of inadvertent doping were discussed through real-life examples, such as in the case of athlete Brandon Copeland, who faced career-altering consequences despite taking precautions. The importance of third-party testing programs, such as NSF Certified for Sport and Informed Sport, was highlighted to ensure the safety and purity of supplements. Additionally, the unit addressed potential risks of supplement use, including contamination, adverse health effects, and interactions with medications. The idea that athletes should prioritize a solid nutritional foundation before resorting to dietary supplements was reinforced and repeated throughout the unit to consolidate knowledge. Lastly, the unit provided guidelines from Health Canada to help athletes use dietary supplements safely and minimize associated risks (Figure 5).

The learning outcomes of the fourth unit were to:

- Describe the risks associated with dietary supplement use.
- Make informed decisions about current and future dietary supplement use.
- Critically evaluate the claims made by dietary supplement products.
- Use WADA, and other academic resources to find information about dietary supplements.

The Theory of Planned Behaviour constructs addressed in Unit 4 were attitudes, injunctive norms, descriptive norms, and perceived behavioral control.

#### 4.4.2. Program Integration into CourseLink

Following content development, the research team collaborated with the Open Learning and Educational Support (OpenEd) department at the University of Guelph, who converted the content into online, self-directed modules via CourseLink, the University of Guelph’s learning management system. CourseLink was selected as an online platform for several reasons. First, it is easily accessible by all students, and they are already familiar with it. Additionally, CourseLink allows tracking of participants progress on CourseLink and moderates their access. After an initial meeting, the OpenEd team assigned staff who will provide instructional design and development expertise to ensure that the program meets quality assurance, copyright, and accessibility standards. A Distance Learning Program Development Specialist at OpenEd worked closely with the research team to set up the program on CourseLink. She provided guidance on the structure to be followed facilitating program content to be adapted to this platform.

The program content was run through Turnitin, a plagiarism detector. The OpenEd team confirmed the content was written in lay language and was easily understood. The research team selected several images to be used in each unit, which were then sent to OpenEd for final selection. This process took approximately three months to fully set up the program online.

#### 4.4.3. Program Structure and Content Layout

The program consisted of a course overview page and a course conclusion page, in addition to the four units. Each unit consisted of an introduction and learning outcomes page, the unit content, summary page, and unit resources that the participants could use for further exploration. Participants were required to complete the program in a sequential order.

**Figure 6:**
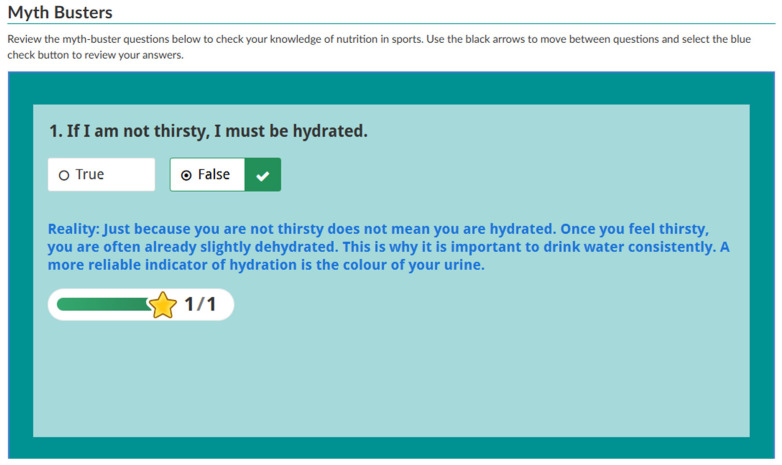
Myth buster—Hydration in Sports unit.

The program incorporated various interactive elements to engage participants. These included fun facts, myth busters, and quizzes. The fun facts provided interesting tidbits related to the course material. Myth busters helped address common misconceptions in an engaging way (Figure 6). Additionally, quizzes were integrated throughout the program to reinforce key concepts.

## 5. Key Theoretical Constructs Addressed in the Program

The program addressed all constructs of the Theory of Planned Behavior through a range of intervention components. However, since the formative assessment revealed that this target population held many misconceptions about dietary supplements, a significant portion of the program was educational and instructive, primarily targeting attitudes. The program influenced beliefs about the perceived advantages of using dietary supplements, clarified the risks associated with their use, corrected misinformation, and emphasized the importance of adopting a food-first approach. Subjective norms were influenced through real-life stories shared by peers whose use of dietary supplements posed a significant risk to their athletic careers. Additionally, injunctive norms were addressed through videos from healthcare professionals, which aimed to reinforce the idea that it is both socially accepted and encouraged to avoid dietary supplements unless medically necessary. Perceived behavioral control was addressed by providing practical resources such as meal planning ideas, food pairing suggestions, and easy-to-follow recipes. Moreover, the program frequently emphasized the importance of consulting healthcare professionals to set personalized goals and make informed decisions, which aimed to empower participants to take control of their decisions.

Table 1 provides an overview of the key theoretical constructs addressed in the program.

## 6. Evaluation

The intervention’s impact was assessed through an online questionnaire, administered to all participants twice—once before and once after the program. The questionnaire was adapted from a previous study that was tested for reliability and validity within the target population [47]. Content validity was examined through expert evaluation, which assessed 12 factors, including relevance, brevity, repetition, order, response options, and bias.

The questionnaire consisted of four main sections:

1. Demographic Information: This section collected data on participants’ age, gender, medical conditions, parents’ or guardians’ education levels, ethnic background, smoking habits, and alcohol consumption.
2. Assessment of Knowledge: This section included multiple-choice and true/false questions about dietary supplements, sports nutrition, hydration, and health. For example, participants were asked, “True or False: Dietary supplements should be taken by all athletes to improve athletic performance and optimize their health.” Another question asked was, “When should protein be ingested to enhance and accelerate glycogen repletion?” with multiple-choice response options.
3. Theory of Planned Behavior Constructs: This section included 20 Likert-scale statements that addressed intention (3 items), attitude (4 items), injunctive norms (5 items), descriptive norms (4 items), and perceived behavioral control (4 items). Statements such as “In the next 6 months, I intend to take or keep taking a dietary supplement to improve my performance and/or general health” (intention) and “My peers/friends think I should use dietary supplements to improve my performance, physical appearance, or general health” (injunctive norm) were rated on a five-point Likert scale (1 = strongly disagree to 5 = strongly agree).
4. A “yes/no” question on whether they have been consuming dietary supplements.

Table 2 summarizes the main outcome variables and measurement instruments used to evaluate the intervention.

The internal reliability of the questionnaire was tested through Cronbach’s α values, which were 0.957 for intention, 0.542 for attitude, 0.831 for injunctive norm, 0.743 for perceived behavioral control, and 0.822 for descriptive norm.

## 7. Intervention Outcomes

The primary purpose of the present manuscript is to describe the theoretical framework, development process, and implementation of the “Nutrition for Athletes: A Focus on Dietary Supplements” intervention. The effectiveness of the intervention was evaluated separately and reported in subsequent peer-reviewed publications [48,49]. The intervention was evaluated using pre- and post-intervention questionnaires administered to 30 varsity athletes participating in the program. Briefly, the intervention was associated with significant improvements in nutrition and dietary supplement-related knowledge [48], as well as significant changes in attitudes, perceived behavioral control, and intentions related to dietary supplement use among varsity athletes. However, no significant changes were observed in descriptive and injunctive norms [49]. Detailed analyses and outcome findings are presented in the corresponding manuscripts.

## 8. Conclusions

This article provided an overview of the development of the *“Nutrition for Athletes: A Focus on Dietary Supplements”* program. Based on the Theory of Planned Behaviour, the program aimed to influence the University of Guelph’s varsity athletes’ knowledge, intentions, attitudes, subjective norms, perceived behavioral control, and use related to sports nutrition and dietary supplements.

While the program demonstrated various strengths, including being theory-based, accessible, written in lay language, and interactive, several limitations should be acknowledged. First, the intervention was developed and evaluated within a single Canadian university and focused exclusively on varsity athletes, which may limit the generalizability of the findings to other populations and sport settings. Second, the intervention relied on self-reported measures, which may be subject to recall and social desirability bias. Another setback was the lack of direct communication between the participants and the research team. As the program was self-paced, participants had no immediate opportunity to ask questions or seek clarification, which could have been beneficial for enhancing understanding. However, a resource section was added to each unit in case participants were interested in reading more. Finally, although most questionnaire constructs demonstrated acceptable internal consistency, the lower Cronbach’s α value for the attitude construct suggests that this scale may benefit from further refinement in future research.

Insights from this paper can be used to guide the development of similar theory-based nutrition and dietary supplement interventions targeting university athletes or younger athletic populations in sport and educational settings.

## Figures and Tables

**Table 1:** Theory of Planned Behaviour constructs addressed in the “Nutrition for Athletes: A Focus on Dietary Supplements” program.

Program Component	Description	TPB Construct Addressed
Educational content on sports nutrition and supplement use	- Provided information about balanced nutrition and the importance of meeting dietary needs through food first. - Provided information on potential health risks, lax regulations, and misconceptions associated with dietary supplement use.	Attitude
Video by a healthcare professional	- Provided expert insights into sports nutrition and dietary supplement use. - Reinforced that healthcare professionals advise against unnecessary supplement use by athletes.	Attitude Injunctive Norm
Myth busters	- Clarified common misconceptions about dietary supplement use.	Attitude
Stories from athletes who used contaminated supplements	- The program included stories from two athletes who unknowingly consumed contaminated dietary supplements, which gave a real-life example of risks associated with supplement use.	Descriptive norm
Meal ideas, food pairing examples, nutrition tips, and simple recipes.	- The program offered practical ideas for meal planning and preparing alternatives to commercial supplements, such as sports drinks.	Perceived behavioral control

**Table 2:** Intervention outcome variables and corresponding measurement instruments.

Outcome Variable	Measurement Instrument	Example Item	Scale
Knowledge	Multiple-choice and true/false questionnaire	True or False: Dietary supplements should be taken by all athletes to improve athletic performance and optimize their health.	Correct/incorrect
Attitudes	TPB Likert-scale questionnaire	I believe that using dietary supplements will improve my performance.	1–5 Likert
Injunctive Norms	TPB Likert-scale questionnaire	My peers/friends think I should use dietary supplements to improve my performance, physical appearance or general health.	1–5 Likert
Descriptive Norms	TPB Likert-scale questionnaire	My teammates or training partners regularly use dietary supplements to improve performance, physical appearance or general health.	1–5 Likert
Perceived Behavioral Control	TPB Likert-scale questionnaire	I have complete control over whether to take or not to take dietary supplements.	1–5 Likert
Intentions	TPB Likert-scale questionnaire	I intend to take or continue taking dietary supplements to improve my performance and/or general health.	1–5 Likert
Supplement Use	Self-report yes/no question	Current supplement use	Yes/No

## Data Availability

The original contributions presented in the study are included in the article. The questionnaire is available upon request from the corresponding author.

## Abbreviations

The following abbreviations are used in this manuscript:

TPB	Theory of Planned Behaviour
WADA	World Anti-Doping Association
